# The Association Between Short Sleep Duration and Metabolic Syndrome: A Case-Control Study

**DOI:** 10.2147/DMSO.S438054

**Published:** 2023-12-20

**Authors:** Amgad Mohamed Elshoeibi, Aishat Akomolafe, Aisha Al-Khulaifi, Omar Metwally, Roudah Al-Khayarin, Abdul Rahman Al Abiad, Latifa Al-Naemi, Tawanda Chivese, Habib Farooqui

**Affiliations:** 1 College of Medicine, QU Health, Qatar University, Doha, 2713, Qatar; 2 Department of Population Medicine, College of Medicine, QU Health, Qatar University, Doha, 2713, Qatar

**Keywords:** Sleep Duration, Metabolic Syndrome, Metabolic Disorder, Obesity, Qatar Biobank

## Abstract

**Purpose:**

Short sleep duration and quality are increasingly common in the Middle East and North Africa (MENA) region and has been linked to metabolic syndrome, which increases the risk of cardiovascular disease and diabetes. This study aimed to examine the link between short sleep duration and metabolic syndrome.

**Patients and Methods:**

We conducted a case-control study using data from Qatar Biobank, with 1000 participants categorized into two groups: less than 7 hours of sleep (n=500) and 7 or more hours of sleep (n=500). Metabolic syndrome was defined using WHO criteria, and logistic regression analysis adjusted for age and gender.

**Results:**

There was a higher proportion of individuals with MetS in the short sleep duration group compared to the normal sleep duration group (22.8% vs 15.8%, respectively). The multivariable regression showed that short sleep duration was associated with metabolic syndrome (OR 1.91, 95% CI: 1.14–3.20, P=0.014) and having 1–2 components of metabolic syndrome (OR 1.91, 95% CI: 1.14–3.20, P=0.014), particularly in males (OR: 2.30, 95% CI: 1.07–4.94, P=0.032). Being overweight (OR 2.17, 95% CI: 1.30–3.63, P=0.003) was also associated with a shorter sleep duration. BMI was identified as the main contributor to the association between short sleep duration and metabolic syndrome, while diabetes played a minor role.

**Conclusion:**

Short sleep duration was associated with metabolic syndrome in Qatar, particularly in males.

## Introduction

Short sleep duration and quality are becoming highly prevalent, globally, and particularly in the Middle East and North Africa (MENA) region. A recent population-based study from Qatar reported that 54% of the population on average sleep less than 7 hours per day.^1^ Research has also shown that the incidence of important risk factors for sleep deprivation such as increased screen time, long working hours, and long commute times to and from work are on the rise.^2^

The role of sleep in preserving and maintaining metabolic homeostasis is still not fully understood. Adequate sleep is important for physical and mental well-being. However, sleep patterns vary with different factors such as age, gender, obesity (BMI), stress, and chronic diseases.^3^ Evidence suggests that short sleep duration is associated with chronic illness, obesity, and poor overall health of the population.^1^,^3^ It has also been linked to metabolic syndrome. Current evidence suggests that, globally, approximately 20 to 30% of adults have metabolic syndrome.^4^ Metabolic syndrome is a cluster of disorders including hypertension, central obesity, insulin resistance and atherogenic dyslipidemia which increase an individual’s risk of developing atherosclerotic cardiovascular disease and diabetes. It has been suggested that lack of physical activity, obesity, are the main drivers for the development of metabolic syndrome.^5^

The associations between insufficient sleep duration and cardiometabolic variables, such as obesity, hypertension, dyslipidemia, and hyperglycemia, has been increasingly studied during the past twenty years.^6–9^ Several studies in the literature associate inadequate sleeping duration with the occurrence of metabolic syndrome.^9–11^ A recent meta-analysis by Jing Xie et al highlighted a U-shaped association between sleep duration and poor health outcomes, such as diabetes mellitus, hypertension, cardiovascular disease, and obesity.^12^ These findings imply that sleep duration could be a risk factor for metabolic syndrome. However, there is a limited research evidence on the association between short sleep duration and metabolic syndrome from the Middle East and in particular from Qatar.

The aim of this study was to evaluate the association between short sleep duration and metabolic syndrome in Qatar. Additionally, we investigated the association between the different components of metabolic syndrome with short sleep duration.

## Materials and Methods

### Study Design and Setting

This was a case-control study that was conducted between October 2021 and September 2022. Participants’ information were collected by the Qatar Biobank (QBB) (https://www.qatarbiobank.org.qa/). The QBB is a prospective cohort that was initiated in 2012 to collect biological data along with lifestyle and health related information from Qatar’s populace.^13^ QBB participants are aged 18 or older and are Qatari’s or long-term residents in Qatar (lived in Qatar for 15 or more years). The information included demographic data (age, gender, employment), laboratory measurements (high-density lipoprotein [HDL], low-density lipoprotein [LDL], hemoglobin A1C [HbA1C]), medical history (hypertension, dyslipidemia, diabetes), and sleeping duration.

### Cases and Controls

The study sample consisted of 1000 participants: 500 randomly selected cases and 500 randomly selected controls. Cases were classified as per the American Academy of Sleep Medicine and Sleep Research Society’s recommendations.^14^ Cases were defined as those who had, on average, less than 7 daily hours of sleep. Controls were defined as those who had 7 or more daily hours of sleep on average. In the QBB data, the duration of sleep was assessed using a self-reported questionnaire that asked the following question: “In a typical week during the last year, approximately how many hours of sleep did you get in a 24-hour period? (Including naps)”. Participants had the option to answer with the following categories: <5 hours, 5 to <7 hours, 7 to 8 hours, and >8 hours. The first and last two categories were combined for the cases and controls, respectively. The data did not include individuals suffering from any of the following: (1) history of heart attack (2) history of stroke (3) diagnosed sleeping disorders.

### Metabolic Syndrome

Our study utilized the WHO criteria to define metabolic syndrome.^15^ Participants were deemed to have metabolic syndrome if they had three or more of the listed criteria: (1) Obesity defined as BMI >30 kg/m^2^; (2) dysglycemia defined as HbA1C >5.7% (39 mmol/mol) or previously diagnosed diabetes; (3) self-reported hypertension; (4) raised plasma triglycerides defined as triglycerides ≥1.7mmol/L (5) low HDL-cholesterol defined as HDL <0.9 mmol/L and <1.0 mmol/L in men and women, respectively. Participants were categorized into three groups based on their metabolic syndrome status: no risk factors for metabolic syndrome, 1–2 risk factors, and metabolic syndrome. It is important to note that the original criteria uses raised blood pressure, however due to a lack of data on blood pressure we utilized self-reported hypertensive status instead. Participants were considered to have diabetes if they had a self-reported history of diabetes or their HbA1C levels was ≥6.5% (48 mmol/mol). Prediabetes was also defined using HbA1C between 5.7% to <6.5% (39 to <48 mmol/mol). These cutoffs were based on the American Diabetes Association guidelines for diabetes diagnosis.^16^ Participants were deemed to have dyslipidemia if one or more of the following was present: self-reported history of a dyslipidemia diagnosis; total cholesterol ≥5.2 mmol/L; LDL-cholesterol ≥3.4 mmol/L; low HDL-cholesterol defined as HDL <0.9 mmol/L and <1.0 mmol/L in men and women, respectively; raised plasma triglycerides defined as triglycerides ≥1.7mmol/L.^17^

### Other Data

Age was categorized into <30, 30 to <40, 40 to <50 and ≥50 years old. Body mass index (BMI) was calculated by dividing weight (in kilograms) by height (in meters squared). BMI was categorized into underweight (<18.5), normal (18.5 to <25), overweight (25 to <30), and obese (≥ 30) based on the WHO criteria.^18^ However, for the purpose of analysis it was categorized into three groups: not overweight/obese (BMI < 25), overweight (25 ≤ BMI < 30), and obese (BMI ≥ 30) due to the limited number of participants in the underweight group. Waist circumference was measured using a tape in the horizontal plane midway between the lowest ribs and the iliac crest. Nationality was recorded as a categorical variable with two levels: Qatari and Non-Qatari. Smoking status was recorded as a categorical variable with three levels: non-smokers, smokers, and missing. Education level was categorized into three groups: primary and secondary, undergraduate, and postgraduate. Employment status was categorized into two groups: employed and unemployed.

### Statistical Analysis

Histograms were used to assess the normality of continuous variables. Normally distributed variables were summarized using means and standard deviation and skewed variables were summarised using median and interquartile ranges (IQR). Student’s independent groups *t*-tests was used to compare groups for normally distributed numerical data, and the Wilcoxon Rank Sum test was used for skewed data. Categorical variables were reported as numbers and percentages. Chi-square test was used to compare categorical variables between cases and controls.

Multivariable logistic regression was used to assess the association between metabolic syndrome and sleep duration. This was also repeated for the components of metabolic syndrome separately. In each model, the outcome was the sleep duration, coded as cases and controls, the exposure was MetS, coded as a categorical variable into these classifications; No MetS, 1 or 2 components of MetS, MetS, with class No MetS as the base category. These models, we adjusted for age and gender as confounders based on our literature search. After each model, we tested model specification using test the link test, and model fit using area under the ROC. The odds ratio (OR), its 95% confidence interval (95% CI), and exact p-values were reported and interpreted as evidence against the null hypothesis. Stata 17 was utilized to conduct the statistical analysis.

## Results

### Characteristic of Participants

A total of 1000 participants were included in the study, of which, 500 had short sleep and 500 had good sleep. In both groups, the majority of the participants were of Qatari nationality, and this was fairly similar across both groups (77.6% vs 73.4% in controls and cases, respectively). The median age was higher in the individuals with short sleep compared to the individuals group (39.5 vs 35, respectively, p <0.001). Individuals with good sleep had a greater proportion of females (59.4% vs 49.0%, respectively p <0.001) and a lower proportion of unemployed individuals (27.0% vs 41.2%, respectively p<0.001). Moreover, the short sleep group had a higher proportion of overweight and diabetic individuals. There were no differences in smoking status, hypertension, dyslipidemia, LDL, HDL, total cholesterol and triglyceride levels between both groups. The baseline characteristics for both groups are summarized in Table 1.

**Table 1:** Comparison of Baseline Characteristics Between the Good and Poor Sleep Groups

Factor	Level	Good Sleep (N= 500)	Poor Sleep (N=500)	p-value
Age, median (IQR)		35.0 (27.0, 48.0)	39.5 (30.5, 50.0)	<0.001
Gender	Female	297 (59.4%)	245 (49.0%)	<0.001
	Male	203 (40.6%)	255 (51.0%)	
Nationality	Non-Qatari	112 (22.4%)	133 (26.6%)	0.12
	Qatari	388 (77.6%)	367 (73.4%)	
Educational Level	Primary & Secondary	221 (44.2%)	201 (40.2%)	0.27
	Undergraduate	256 (51.2%)	267 (53.4%)	
	Postgraduate	23 (4.6%)	32 (6.4%)	
Employment Status	Unemployed	206 (41.2%)	135 (27.0%)	<0.001
	Employed	292 (58.4%)	365 (73.0%)	
	Missing	2 (0.4%)	0 (0.0%)	
Metabolic Syndrome	No Risk Factors for MetS	93 (18.6%)	55 (11.0%)	<0.001
	1–2 Risk Factors for MetS	328 (65.6%)	331 (66.2%)	
	Metabolic Syndrome	79 (15.8%)	114 (22.8%)	
BMI	Not Overweight/Obese	125 (25.0%)	81 (16.2%)	0.002
	Overweight	197 (39.4%)	234 (46.8%)	
	Obese	178 (35.6%)	185 (37.0%)	
Smoking Status	Non-smokers	392 (78.4%)	395 (79.0%)	0.96
	Smokers	93 (18.6%)	93 (18.6%)	
	Missing	15 (3.0%)	12 (2.4%)	
Hypertension	Non-hypertensive	430 (86.0%)	415 (83.0%)	0.21
	Hypertensive	70 (14.0%)	84 (16.8%)	
	Missing	0 (0.0%)	1 (0.2%)	
Diabetes	No diabetes	346 (69.2%)	305 (61.0%)	0.023
	Prediabetes	80 (16.0%)	97 (19.4%)	
	Diabetes	74 (14.8%)	98 (19.6%)	
Dyslipidemia	No Dyslipidemia	370 (74.0%)	345 (69.0%)	0.080
	Dyslipidemia	130 (26.0%)	155 (31.0%)	
Waist Circumference, mean (SD)		86.0 (12.4)	88.8 (11.8)	<0.001
Triglyceride, median (IQR)		1.1 (0.8, 1.5)	1.1 (0.8, 1.6)	0.56
LDL-Cholesterol, median (IQR)		2.8 (2.2, 3.3)	2.8 (2.3, 3.4)	0.19
HDL-Cholesterol, median (IQR)		1.4 (1.1, 1.7)	1.3 (1.1, 1.6)	0.27
Total Cholesterol, median (IQR)		4.7 (4.1, 5.4)	4.8 (4.2, 5.5)	0.35

**Abbreviations**: IQR, interquartile range; BMI, body mass index; SD, standard deviation; LDL, low-density lipoprotein; HDL, high-density lipoprotein.

### Prevalence of Metabolic Syndrome by Sleep Duration

**Figure 1:**
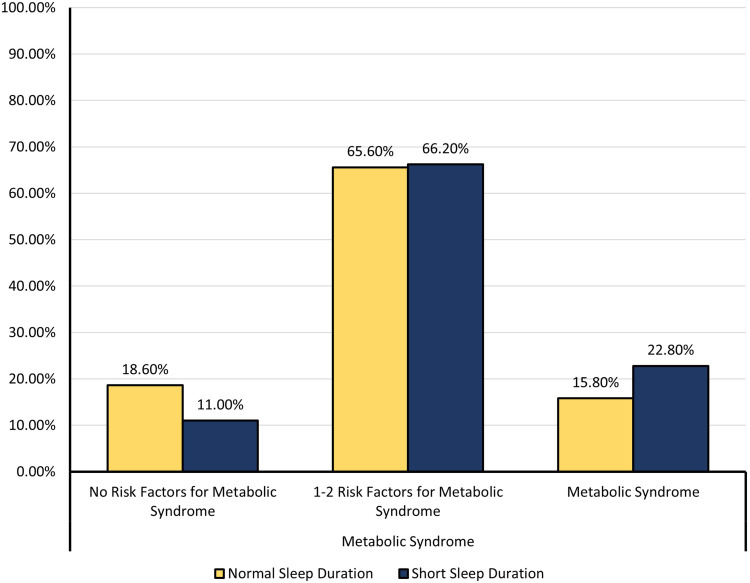
Comparison of proportions of MetS between individuals with good and poor sleep.

Figure 1 is a clustered bar chart comparing MetS by sleep duration categories. There was a higher proportion of individuals with MetS in the short sleep group compared to the normal sleep group (22.8% vs 15.8%, respectively). On the other hand, the good sleep group had a higher proportion of individuals with no risk factors for metabolic syndrome compared to controls (18.6% vs 11.0%, respectively). In the 1–2 risk factors category, both the poor and good sleep groups had similar proportions (65.6% vs 66.2% in the good sleep group and the short sleep group, respectively).

### Association Between MetS and Short Sleep Duration Multivariable Logistic Regression

Table 2 summarizes the results of the multivariable logistic regression. After adjustment for age and gender, individuals with metabolic syndrome had almost 2-fold greater odds of short sleep duration when compared to those without risk factors for metabolic syndrome and with strong evidence against the null hypothesis (OR 1.91, 95% CI: 1.14–3.20, p=0.014). Having 1 or 2 components of metabolic syndrome only also showed an association with short sleep, albeit with a lower OR than that observed for MetS (OR: 1.50, 95% CI: 1.01–2.20; p=0.042).

**Table 2:** Association Between MetS and Its Components with Sleeping Status

Variable	Sublevel	Unadjusted		Overall Adjusted	
		OR (95% CI)	P-value	OR (95% CI)	p-value
Metabolic Syndrome	No Risk Factors	Reference			
	1–2 Risk Factors	1.71 (1.18–2.46)	0.004	1.50 (1.01–2.20)	0.042
	Yes	2.44 (1.57–3.79)	0.000	1.91 (1.14–3.20)	0.014
Dyslipidaemia	No	Reference			
	Yes	1.28 (0.97–1.68)	0.080	1.04 (0.77–1.41)	0.792
Diabetes	No Diabetes	Reference			
	Prediabetes	1.38 (0.99–1.92)	0.061	1.19 (0.83–1.70)	0.336
	Diabetes	1.50 (1.07–2.11)	0.019	1.29 (0.88–1.88)	0.189
Hypertension	No Hypertension	Reference			
	Hypertension	1.24 (0.88–1.76)	0.215	1.02 (0.70–1.49)	0.917
BMI	Not Overweight/ Obese	Reference			
	Overweight	1.83 (1.31–2.57)	0.000	1.60 (1.12–2.27)	0.009
	Obese	1.60 (1.13–2.27)	0.008	1.38 (0.95–2.01)	0.088
Hypertriglyceridemia	No	Reference			
	Yes	1.32 (0.98–1.77)	0.070	1.06 (0.78–1.46)	0.698
Hypercholesterolemia	Normal	Reference			
	Borderline	1.03 (0.77–1.36)	0.863	0.91 (0.68–1.22)	0.522
	High	0.98 (0.61–1.57)	0.924	0.82 (0.51–1.33)	0.427
Hypo-HDL Cholesterol	No	Reference			
	Yes	1.09 (0.76 −1.56)	0.648	0.86 (0.59–1.26)	0.438
Waist Circumference		1.02 (1.01–1.03)	0.000	1.01 (0.99–1.02)	0.440

**Abbreviations**: OR, odds ratio; CI, confidence interval; IQR, interquartile range; BMI, body mass index; SD, standard deviation; LDL, low-density lipoprotein; HDL, high-density lipoprotein.

Of the individual components of MetS, only being overweight was significantly associated with higher odds of short sleep duration (OR: 1.60, CI: 1.12–2.27, p=0.009). Although the other MetS components showed some weak associations with sleep duration, these had weak evidence against the null hypothesis at the study’s sample size (Table 2).

After stratifying by gender and adjusting for age (Table 3), we found that the association between MetS and short sleep duration was more pronounced in males with strong evidence against the model hypothesis (OR: 2.30, 95% CI: 1.07–4.94, P=0.032) whereas, in females, the association was slightly weaker with weak evidence against the model hypothesis (OR: 1.66, 95% CI: 0.817–3.38, p=0.161). For individuals with 1 or 2 components of metabolic syndrome, males reported a stronger association than females, however, both associations had weak evidence against the model hypothesis. Males who were overweight had 2.17 greater odds of being sleep deprived (95% CI: 1.30–3.63, p=0.003) whereas females in the same category only had 1.20 greater odds of being sleep deprived but with weak evidence against the model hypothesis (95% CI: 0.74–1.95, p=0.460). Similarly, males who were obese had 1.82 greater odds of being sleep deprived (95% CI: 1.05–3.17, p=0.034). This association was absent in female obese participants (OR: 1.09, 95% CI: 0.65–1.81, p=0.743). All other components of metabolic syndrome were not associated with a lower sleep duration (Table 3).

**Table 3:** Association Between MetS and Its Components with Sleeping Status Stratified by Gender

Variable	Sublevel	Female		Male	
		OR (95% CI)	P-value	OR (95% CI)	p-value
Metabolic Syndrome	1–2 Risk factors	1.28 (0.77–2.12)	0.346	1.84 (1.00–3.34)	0.050
	Yes	1.66 (0.817–3.38)	0.161	2.30 (1.07–4.94)	0.032
Dyslipidemia	Yes	1.14 (0.74–1.76)	0.539	0.95 (0.62–1.44)	0.802
Diabetes	Prediabetes	1.09 (0.65–1.84)	0.738	1.23 (0.75–2.04)	0.411
	Diabetes	1.56 (0.94–2.60)	0.088	1.01 (0.58–1.76)	0.984
Hypertension	Hypertension	1.02 (0.59–1.76)	0.954	1.00 (0.59–1.70)	0.997
BMI categories	Overweight	1.20 (0.74–1.95)	0.460	2.17 (1.30–3.63)	0.003
	Obese	1.09 (0.65–1.81)	0.743	1.82 (1.05–3.17)	0.034
Hypertriglyceridemia	Yes	1.38 (0.85–2.25)	0.194	0.85 (0.56–1.29)	0.450
Hypercholesterolemia	Borderline	0.83 (0.56–1.24)	0.367	0.99 (0.64–1.54)	0.981
	High	1.17 (0.58–2.36)	0.655	0.59 (0.30–1.16)	0.128
Hypo-HDL Cholesterolemia	Yes	0.84 (0.36–1.94)	0.682	0.85 (0.55–1.31)	0.456
Waist Circumference in cm		1.00 (0.98–1.02)	0.711	1.01 (0.99–1.03)	0.483

**Abbreviations**: OR, odds ratio; CI, confidence interval; IQR, interquartile range; BMI, body mass index; SD, standard deviation; LDL, low-density lipoprotein; HDL, high-density lipoprotein.

## Discussion

In this case-control study of 1000 participants in the Qatar Biobank aged 18 years and above, we found that short sleep duration was associated with MetS and having 1–2 Mets components. Furthermore, this association was more pronounced in males than in females. We also found that being overweight was strongly associated with shorter sleep, particularly in males but not in females. Additionally, we found that the major contributing component to the observed association between short sleep duration and MetS is BMI. Diabetes was also associated with MetS but with a lesser extent. However, the association between short sleep duration and the other individual components of MetS, such as dysglycemia, hypertension, and dyslipidaemia were weak and non-significant.

We found that MetS was associated with a 2 times increase in the odds of short sleep and having 1–2 MetS components was associated with a 1.50 times increase in the odds of short sleep. These findings are consistent with previously published research literature.^19–21^ A meta-analysis by Che et al found that beginning from 7–8 hours of sleep, for every hour reduction in sleep duration, the risk of metabolic syndrome increases by 8%.^22^ Although the biological basis is still unknown, there are multiple putative immunologic, endocrinologic, and metabolic processes proposed to explain the associations between MetS and lower sleeping durations.

Of the Mets component, being overweight showed the strongest associations with a reduction in sleep duration, with an adjusted odds ratio of 1.60. This finding is corroborated by published research evidence highlighting several pathways to explain this association.^9^,^23^,^24^ According to Iftikhar et al, when sleep is restricted, the levels of ghrelin rise while leptin levels drop which leads to increased hunger especially for fatty and starchy food as these increase the activity of the neuronal reward pathways.^21^ These hormonal fluctuations could result in an increase in BMI. Another possible explanation for the association between BMI and short sleep duration is mechanical obstruction caused by fat around the neck. Although this typically occurs in sleep apnea when the BMI is very high, it still occurs to some extent in individuals who are overweight or obese.^25^,^26^ Finally, behavioral studies suggest that the resulting exhaustion from chronic sleep deprivation could result in a decline in physical activity that leads to an increase in BMI.^27^

We also found that diabetics had a 1.29 times increase in odds of being sleep deprived, but with weak evidence. People with diabetes may experience a decrease in both the amount and quality of sleep as a result of the frequent disruptions caused by manifestations of diabetes such as nocturia and diabetic neuropathic pain.^28^ Another proposed mechanism is that sleep deprivation causes the hypothalamus to secrete corticotrophin-releasing hormone (CRH) which activates the pituitary gland to release the adrenocorticotropic hormone (ACTH). ACTH leads to the secretion of cortisol and catecholamines^21^ resulting in a decrease in adiponectin and an increase in leptin, IL-6, TN-alpha, lipolysis, and insulin resistance.^8^ In short, short sleep duration has been reported to cause diabetes through insulin resistance, a sequela which contributes to the development of metabolic syndrome.^24^

We did not find an association between short sleep duration and the other components of metabolic syndrome (dysglycemia, hypo-HDL cholesterolemia, and Hypertriglyceridemia). However, several other previous studies have previously established such associations.^23^,^29^,^30^ High blood pressure and dyslipidemia are connected to poor sleep hygiene. People who sleep for less than 7 h were shown to have significantly elevated mean SBP compared to people that sleep for more than 7 h.^23^ Short sleep duration has been shown to increase salt retention, heart rate and sympathetic nervous system activity, all of which contribute to a higher blood pressure.^29^ We could not demonstrate these associations which could be attributed to the differences between populations.

We observed that the association between MetS and short sleep duration was more prominent in males, with an adjusted odds ratio of 2.30 in comparison to females (odds ratio of 1.66). These results are similar to those of Kim et al who reported that a sleeping duration of less than 6 hours was associated with MetS only in males while sleep duration.^24^ This could be explained by lifestyle factors such as smoking, drinking alcohol, and consuming caffeine which are more common among men. Furthermore, the sex hormones estrogen and testosterone may also play a role in this difference. Estrogen has been shown to play a role in increasing total sleep duration.^31^ Interestingly, research also shows that postmenopausal women have shorter sleep duration and poorer sleep quality compared to premenopausal women and this could be attributed to the menopausal decline of estrogen.

Due to the categorical nature of the reported sleep durations in our study, we were unable to assess the association between long sleep duration and metabolic syndrome. However, existing literature suggests that the association between short sleep duration and metabolic syndrome might be a U-shaped distribution. A cross sectional study by Kim et al reported an association between long sleep duration (>10 hours of sleep) and MetS. When assessing the components of MetS they reported that increased triglycerides component of MetS was the main contributor to the association in both men and women.^30^ Interestingly this association was seen in both the short and long sleep duration groups in their study. However, other studies conducted by Iftikhar et al, and Liang et al, reported no such associations between long sleep duration and MetS.^20^,^32^ Hence, further research is therefore warranted to further assess the link between long sleep durations and metabolic syndrome.

The findings of our research are important for both clinical practice and public health, particularly in relation to the primary and secondary prevention of metabolic syndrome. Promoting sleep hygiene throughout one’s life may be a valuable strategy for preventing poor cardiometabolic health in men and post-menopausal women.^33^ Our study has found a significant association between short sleep duration and metabolic syndrome. To our knowledge, this paper is one of the few studies to investigate the association between metabolic syndrome and short sleep duration in the Qatari population. Additionally, the reasonable sample size of 1000 participants improves the probability of type 2 error.

Our research has several limitations. Firstly, although this was a case-control study by sampling, measurements were cross-sectional which does not allow us to determine whether short sleep duration causes metabolic syndrome or vice versa. Ideally, prospective cohort studies are needed to establish the temporal relationship between short sleep duration and metabolic syndrome. Secondly, the assessment of sleep duration was based on self-reported data obtained through a questionnaire, which lacks validation against objective measures of sleep duration. However, this question has been used in previous reports from QBB and similar questions have been used to assess similar outcomes to this study.^34–41^ It is important to note that this method of assessment may be subject to recall bias and misclassification of cases and controls. However, it can be assumed that this misclassification is non-differential so would not bias our results but would instead weaken the associations. Adding to that, previous research has demonstrated moderate correlations between self-reported measures of sleep duration and objective measures, suggesting that self-report data can, to some extent, be informative.^42^,^43^ Thirdly, we did not assess the association between sleep quality and metabolic syndrome due to the lack of information on quality of sleep in our study. However, prior research has established a relationship between sleep quality and metabolic syndrome.^44^,^45^ As stated previously, we were also unable to assess the association between long sleep duration and metabolic syndrome. Finally, our study dichotomized sleep duration into short and normal due to the lack of granular data (hourly data) on sleep duration. However, the literature reports a u-shaped association (ie both sleep deprivation and excessive sleep are linked to metabolic syndrome).^22^ Future studies should aim to investigate these relationships further.

## Conclusion

To summarize, our study suggests that short sleep duration is linked to MetS and having 1–2 Mets components, particularly in males. Being overweight or obese is also associated with shorter sleep, particularly in males. BMI was identified as the main contributor to the association between short sleep duration and MetS, while diabetes played a minor role. No significant associations between short sleep duration and other individual components of MetS were found. Finally, literature suggests that it may also be important for future studies to explore the association between long sleep duration and MetS.
